# Opioid-Sparing Anesthesia for Open Total Pancreatectomy and Splenectomy Using the External Oblique Intercostal (EOI) Block: A Case Report

**DOI:** 10.7759/cureus.78815

**Published:** 2025-02-10

**Authors:** Hao Yuan Lim, Chi Ho Chan

**Affiliations:** 1 Department of Anaesthesiology, Sengkang General Hospital, Singapore, SGP; 2 Department of Anaesthesiology, Singapore General Hospital, Singapore, SGP

**Keywords:** analgesia, external oblique intercostal, laparotomy, opioid-free, regional anesthesia, upper abdominal surgery

## Abstract

The external oblique intercostal (EOI) block is a relatively new fascial plane block that had been described for upper abdominal surgery. While it has had relatively good analgesia efficacy in the literature for various upper abdominal surgeries, it has yet to be proven to be effective as the sole analgesia technique. We present a 74-year-old Chinese female patient undergoing an open total pancreatectomy and splenectomy using the bilateral EOI block as the primary analgesia technique. A bilateral EOI block was performed preoperatively using 25 mL of 0.2% ropivacaine, and a catheter was inserted into the EOI plane for postoperative analgesia. No long-acting opioids were used intraoperatively. Twenty-five milliliters of 0.2% ropivacaine was supplemented through the catheter 30 minutes prior to the end of surgery. She emerged from anesthesia and reported only mild discomfort at the postanesthesia care unit (PACU). Postoperatively, the EOI catheters were kept for five days, and the patient only required the addition of regular 1 g of intravenous paracetamol every six hours to maintain effective analgesia. Only two doses of 50 mg intravenous tramadol were administered in the first 12 hours as rescue analgesia. We demonstrated that EOI block can provide effective analgesia for upper abdominal surgery, achieving a significant opioid-sparing effect intraoperatively and opioid reduction during the postoperative period.

## Introduction

Epidural analgesia remains the gold standard for postoperative analgesia after major abdominal surgery; however, this is not without major side effects or risks such as hypotension and may also contribute to reduced mobility, which can hinder recovery. With the advancement of regional analgesia, new motor-sparing techniques have been described in coverage of the upper abdominal wall. The external oblique intercostal (EOI) block was first described in 2019 by Hamilton et al. and evaluated by Elsharkawy et al. to provide analgesia to the dermatomes of the upper abdomen [1,2]. The use of EOI block for upper abdominal surgery has been reported with significant success; however, these reports also combine it with other techniques such as intrathecal morphine or intravenous tramadol as lack of visceral coverage was expected with this block [3-7]. We present a case of a 74-year-old female patient who underwent a total pancreatectomy with segmental pulmonary vein resection and splenectomy for locally advanced head of pancreas carcinoma after neoadjuvant chemotherapy. A bilateral EOI block with catheter insertion was performed before surgery for intraoperative and postoperative analgesia. Minimal supplemental analgesia was required, with no long-acting opioid use intraoperatively, and only two doses of 50 mg intravenous tramadol were given during the entire postoperative period. The EOI block is more effective than what we initially expected, and we believe that the EOI is a viable alternative to epidural analgesia for upper abdominal surgery with similar efficacy and fewer side effects.

## Case presentation

A 74-year-old Chinese female patient, 51.8 kg, 153 cm, BMI 22.1, was electively admitted for a total pancreatectomy with segmental pulmonary vein resection and splenectomy for locally advanced head of pancreas carcinoma after neoadjuvant chemotherapy. She has a medical history of hypertension and hyperlipidemia.

Her surgery was conducted under combined general and regional anesthesia. Prior to the induction of anesthesia, a 20 G intravenous cannula was inserted and standard monitoring was applied. The patient was preoxygenated; induced with 80 mg of propofol, 50 mcg of fentanyl, and 50 mg of atracurium; and intubated with a Portex® #7.5 endotracheal tube (ICU Medical, San Clemente, CA, US) using a direct laryngoscopy and a bougie. After intubation, a 20 G left radial intra-arterial cannula, a right internal jugular vein central venous cannula, a 14 F nasogastric tube, and an esophageal temperature probe were inserted.

**Figure 1:**
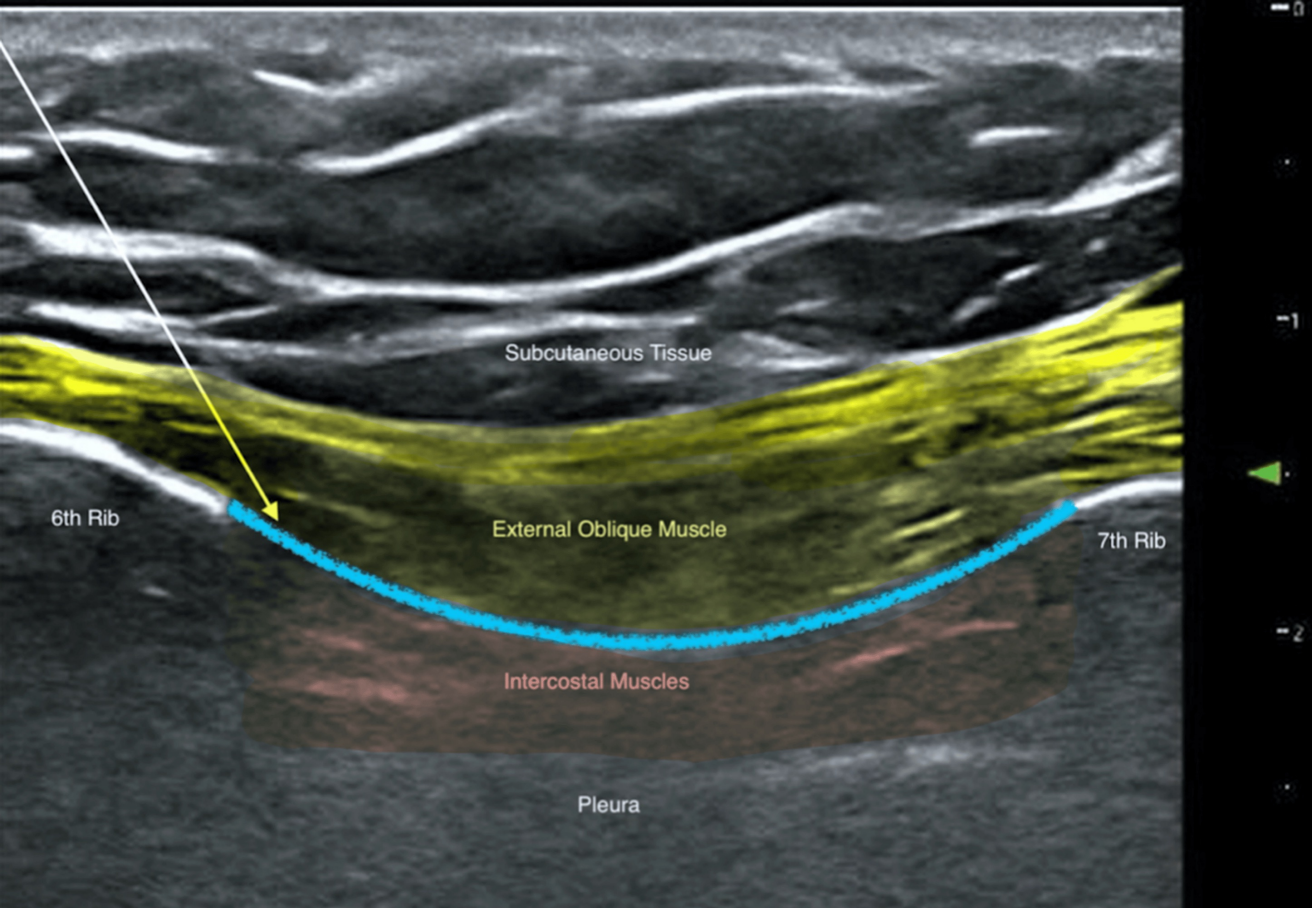
An ultrasound image of the EOI block A linear ultrasound probe was placed in the sagittal oblique plane, with the probe's cranial end rotated slightly medially, at the level of the sixth rib, between the mid-clavicular and anterior axillary lines. The surrounding structures, including the sixth and seventh ribs, subcutaneous tissue, external oblique muscle, and pleura, are labeled. The white arrow indicates the path of the block needle. The yellow highlight outlines the external oblique muscle. The red highlight outlines the intercostal muscles. The blue highlight denotes the fascial plane target for the EOI block. EOI: external oblique intercostal

A bilateral EOI catheter was inserted after the induction of anesthesia using ultrasound guidance. In the supine position, a linear ultrasound transducer was placed in the sagittal oblique plane, with the probe cranial end rotated slightly medially, at the level of the sixth rib, between the mid-clavicular and anterior axillary lines as described by Elsharkawy et al. [2]. An ultrasound image of the EOI block and its surrounding anatomy is shown in Figure 1. Using an in-plane approach, the plane between the external oblique muscle and the intercostal muscle between the sixth and seventh rib was hydrodissected using normal saline, and the catheter was inserted and secured at the 10 cm mark at the skin, with 7 cm inside the EOI plane. After securing the catheter, an analgesic block was performed using 25 mL of 0.2% ropivacaine injected through the catheter.

Intraoperative anesthesia was maintained using inhalational sevoflurane targeted to a minimal alveolar concentration of 0.7-0.8, intravenous atracurium infusion, and intravenous dexmedetomidine with loading of 0.5 mcg/kg over 30 minutes, maintained at 0.3 mcg/kg/hr and stopped one hour prior to the end of surgery. A direct rooftop incision was performed by the surgeon to enter the abdomen, and a total pancreatectomy with splenectomy was performed. The total operation time was six hours and 15 minutes. There was a moderate sympathetic response to the skin incision with a spike in the heart rate from 60 to 78 beats per minute and a rise in blood pressure from 90/58 to 122/74 mmHg, which was resolved with the administration of 50 mcg of fentanyl bolus. The patient remained hemodynamically stable with further sympathetic surges or hypotension due to the anesthesia. Total analgesia given throughout the operation included two doses of 1 g of paracetamol and 150 mcg of fentanyl. An additional 25 mL of 0.2% ropivacaine was injected through the catheter 30 minutes prior to the end of surgery. Intravenous atracurium infusion was stopped one hour before the end of surgery, and neuromuscular blockade was reversed with 2.5 mg of neostigmine and 0.4 mg of glycopyrrolate. The patient was extubated uneventfully.

On arrival at the postanesthesia care unit (PACU), she remained comfortable and only reported mild discomfort. The EOI block was maintained using a CADD-Solis™ pump (ICU Medical, San Clemente, CA, US) programmed to deliver an intermittent bolus of 20 mL of 0.2% ropivacaine on each side every four hours (to start four hours after the last intraoperative dose). The patient was transferred to a high-dependency unit for postoperative monitoring.

On postoperative day (POD) 0, she received one dose of 1 g of intravenous paracetamol and two doses of 50 mg intravenous tramadol during the first 12 hours after surgery for a pain score of 2-3 out of 10. Subsequently, her pain was well managed with intravenous paracetamol and the EOI block. By POD 2, she did not require any other form of intravenous or oral analgesia, and effective analgesia was provided via the EOI block alone. The EOI catheters were removed on POD 4. She did not require any further analgesia after POD 4. The pain score reported by the patient was 2 out of 10 at rest and 6-7 out of 10 on movement on POD 1-3. She was able to walk 22 m on POD 2 assisted by the physiotherapist and improved to up to 110 m by POD 7. She recovered well and was discharged on POD 9.

## Discussion

Major abdominal surgery is associated with a higher risk of postoperative complications, and this is exceptionally higher in surgeries involving the upper abdomen such as esophagectomy, gastrectomy, hepatectomy, and pancreatectomy [8,9]. In addition to the disruption of diaphragmatic function from surgical handling of the abdominal viscera, postoperative pain may hinder respiratory excursion and cause diaphragm splinting. Epidural analgesia has been shown to be superior to systemic opioids for the management of postoperative pain for major abdominal surgery and has been shown to reduce postoperative pulmonary complications [10,11]. However, epidural analgesia is associated with major risks such as dural puncture resulting in intracranial hypotension leading to postdural puncture headache or intracranial bleeding, nerve injury, spinal epidural hematoma, spinal epidural abscess, and inadvertent spinal anesthesia. Moreover, epidural analgesia may also be associated with significant side effects including hypotension, urinary retention, elevated fall risk from lower limb weakness and numbness, itchiness, nausea, and shivering. Unfortunately, preexisting alternative regional anesthesia techniques including the subcostal traditional transversus abdominis plane (TAP) block and the erector spinae plane (ESP) block seem to demonstrate an inferior efficacy for postoperative pain relief [12,13].

The EOI block was evaluated in a cadaveric study by Elsharkawy et al., which demonstrated that the EOI block resulted in the spread of the dye deep to the external oblique and serratus anterior muscles in 100% of the blocks and 75% of the dye spread deep to the pectoris muscle, consistently covering the anterior and lateral cutaneous branches of the intercostal nerves [2]. This was thought to be superior to the TAP block, which may not reliably block the lateral cutaneous nerves to provide anesthesia for the upper lateral abdomen [14,15]. Elsharkawy et al. also performed the EOI block on 22 patients who underwent a variety of major abdominal surgeries, which was able to show consistent blockage of the T6 to T10 dermatomes, with evidence of the anterior and lateral branches of upper thoracoabdominal nerve blockage [2]. As a result, the EOI block is expected to provide analgesic coverage for upper abdominal surgeries.

Multiple cases have been reported on the use of EOI for a variety of major upper abdominal surgeries, but many of these studies did not adequately describe the efficacy of the block or have used additional analgesia that may have masked the true efficacy of the EOI block. In a case series by Liotiri et al., a single-shot EOI block was performed for two patients undergoing laparoscopic hepatic metastasectomy, and an EOI catheter was inserted in one patient for open right hepatectomy [3]. The EOI block provided good analgesia with no postoperative opioids required during the postoperative period. However, the two patients received 200 µg of intrathecal morphine prior to the start of surgery, which may have confounded the interpretation of the efficacy of the EOI block. Lee et al. and O’Donovan and Martin reported the use of EOI catheter insertion for open cholecystectomy with good results but did not report intraoperative opioid use and PACU pain score [6,7]. Lukács and Lőrincz also demonstrated the effectiveness of bilateral EOI block after pancreatic surgery via bilateral subcostal incision, but the block was inserted post operation in the intensive care unit [4]. Somita et al. reported a case of single-shot EOI block for open cholecystectomy, and the patient was comfortable after surgery with a VAS score of 3-4 out of 10 in PACU. However, they also did not document intraoperative opioid use [5].

In our case, we demonstrated that EOI block with simple analgesia may be sufficient to provide adequate analgesia for major upper abdominal surgery in both the intraoperative and postoperative periods. The EOI block demonstrated effectiveness beyond our expectations, as the block was not expected to cover the abdominal visceral innervation. Although additional analgesics, including intravenous paracetamol and low-dose infusion of intravenous dexmedetomidine, were administered intraoperatively, we do not believe these agents alone provided sufficient efficacy to account for the observed analgesia to the visceral organs. Currently, there are no studies that have evaluated the spread of the EOI block posteriorly. We postulate that the EOI block may have potentially spread posteriorly into the paravertebral space to provide anesthesia to the visceral organs. Nonetheless, we believe that the EOI block is a viable alternative to epidural analgesia for open upper abdominal surgery with similar efficacy, is easy to perform in the supine position, and has fewer side effects. Further clinical trials are warranted to study the efficacy of EOI block when compared to the gold standard of epidural analgesia.

## Conclusions

The EOI block has shown evidence in multiple case series that it is able to provide adequate pain relief as part of the multimodal analgesia regime for open upper abdominal surgery. However, we have demonstrated that the EOI catheter with just the addition of simple analgesia provides sufficient analgesia during the intraoperative and postoperative periods. However, further larger studies would be warranted to establish the efficacy of the EOI block when compared to the gold standard of epidural analgesia.

## References

[1] Hamilton DL, Manickam BP, Wilson MA, Abdel Meguid E (2019). External oblique fascial plane block. Reg Anesth Pain Med.

[2] Elsharkawy H, Kolli S, Soliman LM, Seif J, Drake RL, Mariano ER, El-Boghdadly K (2021). The external oblique intercostal block: anatomic evaluation and case series. Pain Med.

[3] Liotiri D, Diamantis A, Papapetrou E, Grapsidi V, Sioka E, Stamatiou G, Zacharoulis D (2023). External oblique intercostal (EOI) block for enhanced recovery after liver surgery: a case series. Anaesth Rep.

[4] Lukács FV, Lőrincz J (2023). # 36480 Bilateral external oblique intercostal catheter for postoperative analgesia after pancreatoduodenectomy via bilateral subcostal incision in a patient with acquired haemophilia A: case report. Reg Anesth Pain Med.

[5] Christopher S, Sahithi R, Gopal TVS, Rajesh KC (2024). External oblique intercostal plane block for open cholecystectomy-a novel technique. J Indian Coll Anaesthesiol.

[6] Lee M, Ayad M, Diz Ferre JL, Oliver LA, Swerchowsky N, Ayad S (2024). Ultrasound-guided external oblique intercostal block as part of multimodal analgesia for unplanned open cholecystectomy. Cureus.

[7] O'Donovan B, Martin B (2021). The novel use of an external oblique nerve catheter after open cholecystectomy. Cureus.

[8] Smetana GW, Lawrence VA, Cornell JE (2006). Preoperative pulmonary risk stratification for noncardiothoracic surgery: systematic review for the American College of Physicians. Ann Intern Med.

[9] Yang CK, Teng A, Lee DY, Rose K (2015). Pulmonary complications after major abdominal surgery: National Surgical Quality Improvement Program analysis. J Surg Res.

[10] Rigg JR, Jamrozik K, Myles PS (2002). Epidural anaesthesia and analgesia and outcome of major surgery: a randomised trial. Lancet.

[11] Salicath JH, Yeoh EC, Bennett MH (2018). Epidural analgesia versus patient-controlled intravenous analgesia for pain following intra-abdominal surgery in adults. Cochrane Database Syst Rev.

[12] Desai N, El-Boghdadly K, Albrecht E (2021). Epidural vs. transversus abdominis plane block for abdominal surgery-a systematic review, meta-analysis and trial sequential analysis. Anaesthesia.

[13] Gao Y, Liu L, Cui Y, Zhang J, Wu X (2022). Postoperative analgesia efficacy of erector spinae plane block in adult abdominal surgery: a systematic review and meta-analysis of randomized trials. Front Med (Lausanne).

[14] Ma J, Jiang Y, Tang S, Wang B, Lian Q, Xie Z, Li J (2017). Analgesic efficacy of ultrasound-guided subcostal transversus abdominis plane block. Medicine (Baltimore).

[15] Chen Y, Shi K, Xia Y, Zhang X, Papadimos TJ, Xu X, Wang Q (2018). Sensory assessment and regression rate of bilateral oblique subcostal transversus abdominis plane block in volunteers. Reg Anesth Pain Med.

